# 2, 3-Dimethylmaleic anhydride (3, 4-Dimethyl-2, 5-furandione): A plant derived insecticidal molecule from *Colocasia esculenta* var. *esculenta* (L.) Schott

**DOI:** 10.1038/srep20546

**Published:** 2016-02-03

**Authors:** Yallappa Rajashekar, Ngaihlun Tonsing, Tourangbam Shantibala, Javagal R. Manjunath

**Affiliations:** 1 Animal Resources Programme, Institute of Bioresources and Sustainable Development, Department of Biotechnology, Govt. of India, Takyelpat, Imphal-795001, Manipur, India; 2 Department of Spice & Flavour Science, Central Food Technological Research Institute, Mysore-570020, India.

## Abstract

The phasing out of methyl bromide as a fumigant, resistance problems with phosphine and other fumigants in stored product beetles, and serious concern with human health and environmental safety have triggered the search for alternative biofumigants of plant origin. Despite the identification of a large number of plants that show insecticidal activity, and the diversity of natural products with inherent eco-friendly nature, newer biofumigants of plant origin have eluded discovery. Using a bioassay driven protocol, we have now isolated a bioactive molecule from the root stock of *Colocasia esculenta* (L.) and characterized it as 2, 3-dimethylmaleic anhydride (3, 4-dimethyl-2, 5-furandione) based on various physico-chemical and spectroscopic techniques (IR, ^1^H NMR, ^13^C NMR and Mass). The molecule proved to be an efficient biofumigant which is highly toxic to insect pests for stored grains even at very low concentration, but has no adverse effect on seed germination. We finally address the potential for this molecule to become a, effective biofumigant.

Since the advent of agriculture, plants have been used for insect pest control and grain protection^1^,^2^,^3^,^4^. Over the last six decades mainly four chemical classes of insecticides and fumigants are being used for insect pest management and grain protection^5^,^6^,^7^. Due to environmental concerns and human health hazards, many insecticides have been banned and replaced by modern insecticides^8^. Further, due to the problem of resistance to insecticides, there is an urgent need for safer alternatives to conventional chemical insecticides for the control of stored-product insect pests, particularly from natural sources. In this scenario, there is an urgent need to develop newer plant derived eco-friendly potent biofumigants^9^.

Many of the plant volatiles and their constituents have indeed been used as potent fumigants against stored grain insect pests^5^,^10^,^11^. Perhaps the most prominent among them is Azadirachtin, a compound extracted from neem (*Azadirachta indica)* which was used as an antifeedant and insect growth regulator. However due to lack of fumigant toxicity to the insects, commercialization of the product was not successful though it finds use in integrated pest management^12^,^13^,^14^. Another compound Rotenone, one of the earliest plant-derived insecticides isolated from the Derris root, was found effective. However, it was found toxic to the mammalian systems and its use as a controller for stored grain pests was not accepted^15^. The synthetic pyrethroids, currently widely used and most successful, were originally derived from the flowers of *Tanacetum cinerariaefolium*^16^,^17^. However, compounds with new mode of action are needed to deal with the problem of resistance and insect selectivity^4^,^18^. Rajashekar *et al*. (2012) reported that Decaleside, a novel natural insecticide isolated from the edible roots of *Decalepis hamiltonii*, targets the gustatory receptors on the tarsi of insect legs^19^. Recent progress in understanding the biology of plant volatile organic compounds additionally offers new strategies for developing selective pest control agents^20^,^21^,^22^,^23^.

*Colocasia esculenta* var. *esculenta* (L.) Schott, commonly called Taro and a member of the Araceae family, is an ancient crop grown throughout the humid tropics for its edible corms and leaves, as well as for its traditional ceremonial uses^24^. It is a potential source of starch which is highly digestible and good dietary carbohydrate alternative especially for diabetic people^25^. Earlier laboratory experiment disclosed a study on the efficacy of its ethanolic extract on maize weevil *Sitophilus zeamais* (Mots)^26^. There are reports on insecticidal activity of bioactive molecules (Lectins) from *C. esculenta* against certain sucking pests^27^ but no information about activity against stored product insect pests. The present study aims to explore the possible use of *C. esculenta* against various stored grain and household pests.

## Results

### Isolation and identification of the biofumgant

**Figure 1:**
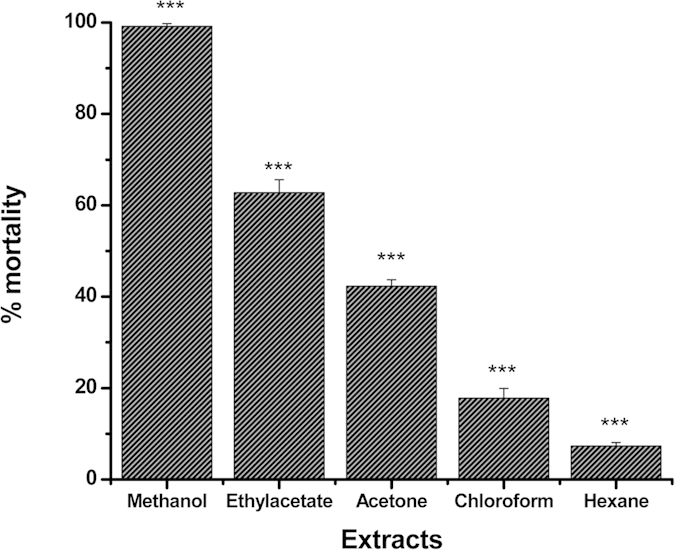
Insecticidal activity of the solvent extracts of *C. esculenta* to *S. oryzae* in the fumigant bioassay. The extracts were applied at 100 μl/l (n = 4, error bars. s.e.m.) one-way ANOVA, ^***^P < 0.001.

**Figure 2:**
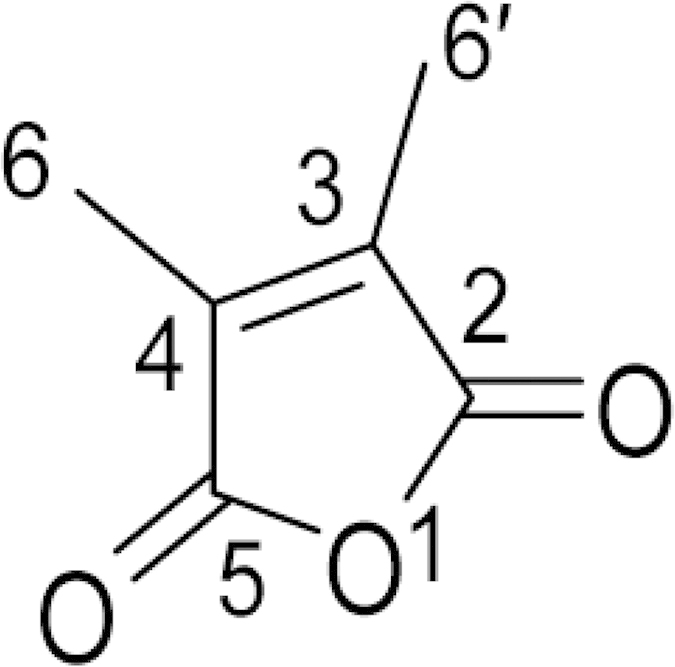
Molecular structure of 2, 3-Dimethylmaleic anhydride (2, 5-Furandione, 3, 4- dimethyl).

The insecticidal activity of the different organic extracts is presented in Fig. 1. Among them, maximum insecticidal activity against the adults of test insects (*S. oryzae*) was shown by the methanol extract, followed by hexane, ethyl acetate, acetone and chloroform extracts. In order to identify the bio active compound which is responsible for the fumigant toxicity, the methanolic extract was subjected to an isolation procedure, which yielded one water soluble bioactive molecule (Fig. S1). The compound was characterized by various physico-chemical and spectroscopic techniques like IR, ^1^H NMR, ^13^C NMR, and GCMS analysis as 2, 3-dimethylmaleic anhydride (3, 4-dimethyl-2, 5-furandione; Fig. 2). In the IR spectrum, the appearance of carbonyl (C=O) band at 1733 cm^−1^ (alkyl stretching expected below 3000 cm^−1^) was in agreement with the conjugated anhydride structure. Mass spectrum showed the molecular ion peak at m/z 126.1, in agreement with its molecular formula. The proton NMR spectrum presented a single peak at 2.07 ppm, which corresponded for the two equivalent methyl groups. The ^13^C NMR spectrum presented three signals at 9.14, 140.47 and 165.83 ppm. The ^13^C attached proton test experiment revealed only 9.14 ppm signal in positive phase confirming the presence of the methyl groups; the other two signals with negative phase are ascribed to quaternary carbon atoms. The signal at 140.47 ppm was assigned to the olefinic carbon atoms at positions 3 and 4, while the signal at 165.83 ppm represented the chemical shift of two carbonyl carbons at positions 2 and 5. The symmetric nature of molecule is responsible for the appearance of only three signals corresponding to six carbons. The spectroscopic studies confirmed the molecule to be 2,3-Dimethylmaleic anhydride (Fig. 2). Physical and spectral data of the compound are presented below.

### 3, 4-Dimethyl-2, 5-furandione

White crystalline solid; yield 0.38%; b.p. 139 °C (lit. bp 223, mp 93–96); ^1^H NMR (500 MHz, CDCl_3_) δ (ppm): 2.07 (singlet, 6H, 2 × CH_3_); ^13^C NMR (125 MHz, CDCl_3_) δ (ppm): 165.83 (C-2, C-5), 140.47 (C-3, C-4), 9.15 (C-6, C-6′). Mass spectrum M^+^ at m/z 126.11.

### Insecticidal activity

The insecticidal activities of crude extracts are shown in Fig 1. Among all the crude extracts, the methanol extract showed maximum activity and was significantly different (Fig. 1). The isolated molecule 2, 3-dimethylmaleic anhydride showed potent insecticidal activity in fumigation bioassay against several insect species, viz., adults of houseflies, cockroaches and stored-product insects (Table 1). Its fumigant toxicity was comparable to that of chemical fumigants for various insect species (Table 2). The insect toxicity was more potent than those of the available natural fumigants except coumaran (Table 3).

In another experiment, 2, 3-dimethylmaleic anhydride was found to be highly toxic to *M. domestica, P. americana, R. dominica, S. oryzae* and *T. castaneum*. Mortality was recorded as 85–98% at a dosage of 100 μg/l in 24 h exposure, whereas 100% mortality was achieved in 72 h exposure (Table 4). Generally, an extended exposure period of 72 h increased the mortality in the species. The results of grain protection showed that the compound caused significant reduction in F1 progeny with increase in exposure period (Table 5).

### Effect on seed germination

The percentage of germination of wheat and maize seeds in 50 and 100 μg/l dosages for different exposure periods (24, 72 h) ranged from 90.4 to 92.1% and 92 to 93.8% respectively (Fig. S2) when compared to respective controls (95–96.2%).

## Discussion

Several chemical and natural insecticides are neurotoxic acting on the central nervous system such as the membrane ion channels (DDT, pyrethroids and Decaleside), on acetylcholinesterase (organophosphate and carbamate), on receptors of neurotransmitters (avermectins and neonicotinoids). Even though these chemicals have brought with them undesired environmental and health problems, they are being used extensively^28^,^29^,^30^,^31^. Similarly, the recently introduced Diamides, found effective on various pests, acts on the ryanodine receptor of nervous system^32^,^33^,^34^. Since the pests continue to evolve resistance to the various compounds which are currently in use, an effective new alternative compound is in urgent need^7^,^35^,^36^.

The volatile molecule 2, 3-Dimethylmaleic anhydride (yield 0.38%) isolated from the root stock of *C. esculenta* was found to be toxic to a variety of insect species when it is used as fumigant. Further, our results clearly showed that treatment of grains with the compound at 100 μg/L caused significant reduction in F1 progeny in all three species and the mortality was increased with extended exposure period in all species (Table 5). The LC_50_ value of allyl acetate was 15 mg/l with *S. oryzae* at 48 h exposure period^37^. The currently used grain fumigants methyl bromide and phosphine have fumigant toxicity (24 h) against *S. zeamais* adults with LC_50_ values 0.67 and 0.006 mg/l^38^. Earlier our studies on Coumaran, a biofumigant molecule isolated from *Lantana camara,* identified it to be toxic to adults of *S. oryzae, C. chinensis* and *T. castaneum* with LC_50_ values 0.45, 0.38 and 0.27 μg/l respectively on 24 h exposure period^5^. The mustard oil major product allylisothiocyanate (AITC) exhibited remarkable activity against grain insect pest *S. oryzae* (adult stage), while adults of the rice weevil are killed after 24 h exposure at less than 6.3 μL/L^39^. The toxicity of 2, 3-Dimethylmaleic anhydride to the rice weevil is comparable to those of methyl bromide and coumaran. Our results clearly demonstrated that the insecticidal potency of 2, 3-Dimethylmaleic anhydride is as good as the other available biofumigants (Table 2). The compound showed potent fumigant activity against various insects including stored-product insects. Although several natural compounds have been reported to exhibit fumigant toxicity, there is no comparative study of the toxicity of a natural compound with that of synthetic insecticides on insects in a fumigant bioassay. The toxicity of 3, 4-Dimethyl-2, 5-furandione to various species was similar to that of other chemical fumigants.

Arannilewa and Odeyemi^26^ reported on the evaluation of insecticidal activity of *C. esculenta* plant on *S. zeamais,* pests of stored maize. The plant material may be highly active if applied at higher concentrations. Further, it was concluded that lectins from these plants had detrimental effect on the growth and development of the insect and may have potential in crop management^40^. *C. esculenta* tuber agglutinin (CEA) may act as a potent insecticidal agent for pest control^41^. We have now identified and characterized a bioactive compound (2, 3-Dimethylmaleic anhydride) from the root stock of *C. esculenta* which acts as a fumigant. Further, the study reveals the main advantage of the plant products as they are less toxic to human beings and qualify for their grain protectant ability amongst low resources farmers who store grains for consumption and planting. The effectiveness of bioactive compounds as insecticides against stored grain and house hold pests has been studied, and these pests have shown susceptibility to plant-derived chemicals. Among them, plant volatile organic compounds are typically volatile and rather lipophilic compounds that can penetrate into insects rapidly and interfere with their physiological functions. Their mechanism of action is not understood at this time. Although the plant volatile compound tested here has activities comparable to chemical fumigants including phosphine and dichlorovos, it is possible that single plant volatile compound may have sufficient potencies to replace the more problematic fumigants and insecticides. There are plenty of literatures available devoted to the insecticidal properties of plant volatile organic compounds and these are indicative of current attitudes and desire to find potentially safer, yet effective, pest management strategies^42^. Further investigations are needed to increase our understanding of the effective use of these technologies.

In conclusion the biofumigant molecule 2, 3-Dimethylmaleic anhydride (3, 4-dimethyl-2, 5-furandione) isolated from the root stock of *C. esculenta* is toxic to various stored grain insect pests and house fly. The lack of adverse effect of the molecule on seed germination makes it highly desirable for grain protection against stored-product insect pests.

## Methods

### Insects

The stored product insects lesser grain borer (*Rhyzopertha dominica)* and rice weevil (*Sitophilus oryzae* L.) were reared on whole wheat, and the rust-red flour beetle (*Tribolium castaneum* Herbst.) on wheat flour with 5% yeast; the pulse beetle (*Callosobruchus chinensis*) was reared on whole green gram as described elsewhere^43^. Housefly (*Musca domestica)* larvae were reared in a mixture of sterilized bran, milk powder and water, and the adults were allowed free access to water and thick paste of condensed milk and milk powder^44^. The American cockroach (*Periplaneta americana*) was reared in plastic tubes with harborages, containing broken wheat and biscuits, and water was provided ad libitum. The cockroaches and housefly were maintained at 23.6 ± 2.5 °C, 70% relative humidity and a photoperiod of 12:12 (Light: Dark).

### Isolation

Using a bioassay-driven procedure, the insecticidal (biofumigant) compound from the methanolic extract of root stock of *C. esculenta* was isolated by three rounds of fractionation on a silica gel column chromatography (Fig. S1). Based on NMR and MS data, the structure of the purified compound was determined (see Supplementary methods).

### Insecticidal activity

The insecticidal activity of extracts of *C. esculenta* against adults of *S. oryzae* was studied by fumigation. Fifty adult insects of known age were released into 0.85-l desiccators that served as the fumigation chambers. In each desiccator, a Whatman No. 1 filter circle (9 cm size) was placed to serve as an evaporating surface for injecting the active extract. For each species, there were four replicates for each dose of the active extract, with equal number of untreated control replicates.

The insecticidal activity of the isolated compounds was tested by fumigation on several insect species, house fly (*M. domestica*), cockroach (*P. americana*) and stored-product insects (*R. dominica, S. oryzae, T. castaneum* and *C. chinensis*). Fifty insects for each treatment were used for all the species except in the case of house fly, where 30 individuals per desiccator were used. The concentration ranged from 0.05 to 2 μl/l, and the effective dosages were chosen based on trial experiments. Four replicates were used for each dosage and LC_50_ values were determined from dose response data using probit analysis^45^.

Another experiment was designed for the mixed age culture (details are given in supplementary information) of different stored grain insect species wherein these were exposed to the purified compound of *C. esculenta* for 24 h and 72 h at 25 ± 2 °C. The mixed age cultures of the insects were weighed in 50 g aliquots into cloth bags (20 cm × 14 cm size) and bags were placed individually in 0.85-l desiccators that served as the fumigation chambers. In each desiccator, a Whatman No. 1 filter circle (9 cm size) was placed to serve an evaporating surface for injecting purified compound; an equal number of untreated control desiccators were maintained. At the end of the exposure, the test insect bags were taken out of the desiccators. The contents of the bags were transferred to individual bottles (12 cm × 5 cm size) and kept at the rearing temperature and humidity conditions for 8 weeks. The insects, which emerged from wheat (*S. oryzae* and *R. dominica*) or survived as adults (*T. castaneum, C. ferrugineus* and *O. surinamensis*) in their respective media were checked at weekly intervals for 8 weeks. Similarly, counts were made in untreated control batches every week. Percent mortality was determined by using the Abbott formula^46^. Percentage reduction in adult emergence of F1 progeny or inhibition rate (%IR) was calculated as


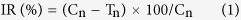


where C_n_ is the number of newly emerged insects in the untreated jar and T_n_ is the number of insects in the treated jar^47^.

In order to compare the insect toxicity of 2,3-Dimethylmaleic anhydride with that of chemical fumigants and natural fumigants, the LC_50_ values were determined for allyl acetate, ethyl formate, carbonyl sulfide and ethylene dichloride by using fumigation bioassay. The details of fumigation bioassay procedure are mentioned in supplementary methods^5^,^43^,^48^.

### Effect on seed germination

Wheat and maize grains were treated with 2,3-Dimethylmaleic anhydride at 50 and 100 μg/l and germination tests were done at 24 h and 72 h of exposure treatment. Fifty seeds from each treatment were randomly selected from each group, soaked in distilled water for about 30 min, kept on filter paper (Whatman No. 1) in a petri dish, moistened daily with distilled water, and allowed to germinate at room temperature (25 ± 2 °C). After 5 d, germinated seeds were counted and percentage germination was calculated^49^.

### Statistical analysis

LC_50_ were determined by Probit analysis^45^. The data were analyzed using One-Way ANOVA (p < 0.05) by Newman-Keuls test using Statplus 2007 software and computer program SAS (version 6.12, SAS Institute Inc. Cory, NC, USA).

## Additional Information

**How to cite this article**: Rajashekar, Y. *et al*. 2, 3-Dimethylmaleic anhydride (3, 4-Dimethyl-2, 5-furandione): A plant derived insecticidal molecule from *Colocasia esculenta* var. *esculenta* (L.) Schott. *Sci. Rep.* **6**, 20546; doi: 10.1038/srep20546 (2016).

## Supplementary Material

Supplementary Information

## Figures and Tables

**Table 1:** Insecticidal activity of purified compound (2, 3-Dimethylmaleic anhydride) against adults of household and stored- product insects by fumigant toxicity.

Insect species	LC50a,^b^	LC_90_	Slope ± SE	Degrees of freedom
*P. americana*	**16.0** (13.5–18.6)	28.8	0.642 ± 0.080	5
*M. domestica*	**2.5** (1.7–3.2)	4.5	0.611 ± 0.141	5
*C. chinensis*	**2.2** (2.0–2.5)	3.96	0.734 ± 0.076	5
*T. castaneum*	**3.4** (2.6–4.5)	6.12	0.371 ± 0.030	5
*S. oryzae*	**3.9** (2.9–0.5)	7.02	0.729 ± 0.081	5
*R. dominica*	**3.6** (2.8–4.2)	6.48	0.825± 0.115	5

^a^LC_50_ and LC_90_ = μg/l.

^b^Values in parenthesis represent confidence limits (n = 6).

**Table 2:** Comparison of insecticidal activity of 2, 3-Dimethylmaleic anhydride with the chemical fumigants.

Insecticides (Fumigants)	LC_50_(μg/l)		
	*Rhyzopertha dominica*	*Tribolium castaneum*	*Sitophilus oryzae*
**2, 3-Dimethylmaleic anhydride**	**3.6**	**3.4**	**3.9**
Ethylene dichloride	0.53	0.70	0.42
Allyl acetate	0.56	1.09	0.91
Ethylformate	0.65	1.12	0.72
Phosphine	8.0	9.8	7.8
Methyl bromide	4.0	8.4	3.6
Dichlorvos	0.03	0.09	0.062

^a^LC_50_ = μg/l.

**Table 3:** Comparison of insecticidal activity of 2, 3-Dimethylmaleic anhydride with the natural fumigants.

Insecticides (Fumigants)	LC_50_(μg/l)		
	*Rhizopertha dominica*	*Tribolium castaneum*	*Sitophilus oryzae*
**2, 3-Dimethylmaleic anhydride**	**3.6**	**3.4**	**3.9**
Carvacrol	15.3	17.0	14.2
1,8 Cineole	33.0	47.0	31.0
Eugenol	11.6	37.1	23.7
Linalool	27.7	49.8	39.2
*α-Pinene*	21.7	61.7	54.9
Coumaran	0.61	3.54	0.85

**Table 4:** Mortality (%) of mixed-age cultures of stored-product insects exposed for 24 h and 72 h to purified compound (2, 3-Dimethylmaleic anhydride) of *C. esculenta.*

Dosage (μg/l)	% Mortality (Mean±SE)^*^					
	*R. dominica*		*S. oryzae*		*T. castaneum*	
	24 h	72 h	24 h	72 h	24 h	72 h
0 (Control)	3.7 ± 1.1^a^	4.9 ± 1.2^a^	2.7 ± 0.6^a^	3.3 ± 0.2^a^	1.5 ± 0.5^a^	2.2 ± 0.8^a^
10	14.7 ± 2.3^b^	28.3 ± 2.6^b^	17.7 ± 1.4^b^	24.3 ± 2.6^b^	15.5 ± 1.5^b^	23.2 ± 2.2^b^
25	34.7 ± 4.1^c^	48.3 ± 2.4^c^	30.7 ± 3.1^c^	46.3 ± 3.6^c^	35.5 ± 3.6^c^	40.2 ± 2.8^c^
50	64.5 ± 3.7^d^	78.9 ± 4.7^d^	58.3 ± 2.4^d^	72.9 ± 4.8^d^	55.8 ± 1.9^d^	75.1 ± 2.6^d^
75	84.7 ± 4.6^e^	92.3 ± 2.6^e^	77.7 ± 2.1^e^	90.3 ± 1.6^e^	69.5 ± 4.5^e^	83.2 ± 2.8^e^
100	98.6 ± 3.5^f^	100^f^	94.5 ± 1.8^f^	100 ^f^	84.8 ± 3.9^f^	100^f^

*There were 5 replicates per dose and in untreated controls (50 g infested media per replicate tested). Values followed by different letters within the vertical columns are significantly different (P < 0.05) by Newman-Keuls test.

**Table 5:** Grain protection potential of 2, 3-Dimethylmaleic anhydride.

Dosage (μg/L^1^)	% Reduction in F1 progeny*					
	*S. oryzae*		*T. castaneum*		*R. dominica*	
	24 h	72 h	24 h	72 h	24 h	72 h
0 (Control)	1.1 ± 0.2^a^	3.1 ± 0.4^a^	2.0 ± 0.4^a^	2.8 ± 0.2^a^	8.9 ± 1.2^a^	6.9 ± 2.4^a^
10	13.1 ± 2.1^b^	24.1 ± 1.4^b^	28.1 ± 2.9^b^	38.2 ± 1.9^b^	15.9 ± 4.7^b^	20.4 ± 2.7^b^
25	35.3 ± 5.1^c^	53.9 ± 1.5^c^	40.6±3.4^c^	50.6±5.4^c^	37.4 ± 5.1^c^	47.4 ± 7.2^c^
50	62.3 ± 2.1^d^	77.3 ± 2.1^d^	55.3 ± 6.6^d^	65.5 ± 3.6^d^	62.4 ± 5.6^d^	82.7 ± 2.6^d^
75	74.5 ± 2.4^e^	90.5 ± 4.4^e^	63.1 ± 1.9^e^	80.2 ± 6.9^e^	75.3 ± 1.4^e^	91.3 ± 2.4^e^
100	90.5 ± 6.1^f^	100^f^	77.5 ± 3.9^f^	100^f^	86.3 ± 3.4^f^	100^f^

Adult emergence in F1progeny of stored product insects from treated grain.

*There were 5 replicates per dose and in untreated controls (50 g infested media per replicate tested). Values followed by different letters within the vertical columns are significantly different (P < 0.05) by Newman-Keuls test.
