# Supplementary material for: 2, 3-Dimethylmaleic anhydride (3, 4-Dimethyl-2, 5-furandione): A plant derived insecticidal molecule from Colocasia esculenta var. esculenta (L.) Schott
Source: Sci Rep. 2016 Feb 3;6:20546. doi: 10.1038/srep20546 (PMC4738332; doi:10.1038/srep20546)
Supplement: Supplementary Information [file srep20546-s1.pdf]

SUPPLEMENTARY INFORMATION

2, 3-Dimethylmaleic anhydride (3, 4-Dimethyl-2, 5-furandione): A plant derived insecticidal molecule from *Colocasia esculenta* var. *esculenta* (L.) Schott

Yallappa Rajashekar[1]*, NgaihlunTonsing[1], Tourangbam Shantibala[1] & Javagal R Manjunath[2],

[1] *Animal Resources Programme, Institute of Bioresources and Sustainable Development, Department of Biotechnology, Govt. of India, Takyelpat, Imphal-795001, Manipur, India*

[2] *Department of Spice & Flavour Science, Central Food Technological Research Institute, Mysore-570020*

*Corresponding author. Tel.: + 91 0385 2446122; Fax: +91 385 2446121.

Correspondence and requests for materials should be addressed to YR (rajacftri@yahoo.co.in)

Supplementary Figures Page No. 3-4

Supplementary Methods Page No. 5 - 8

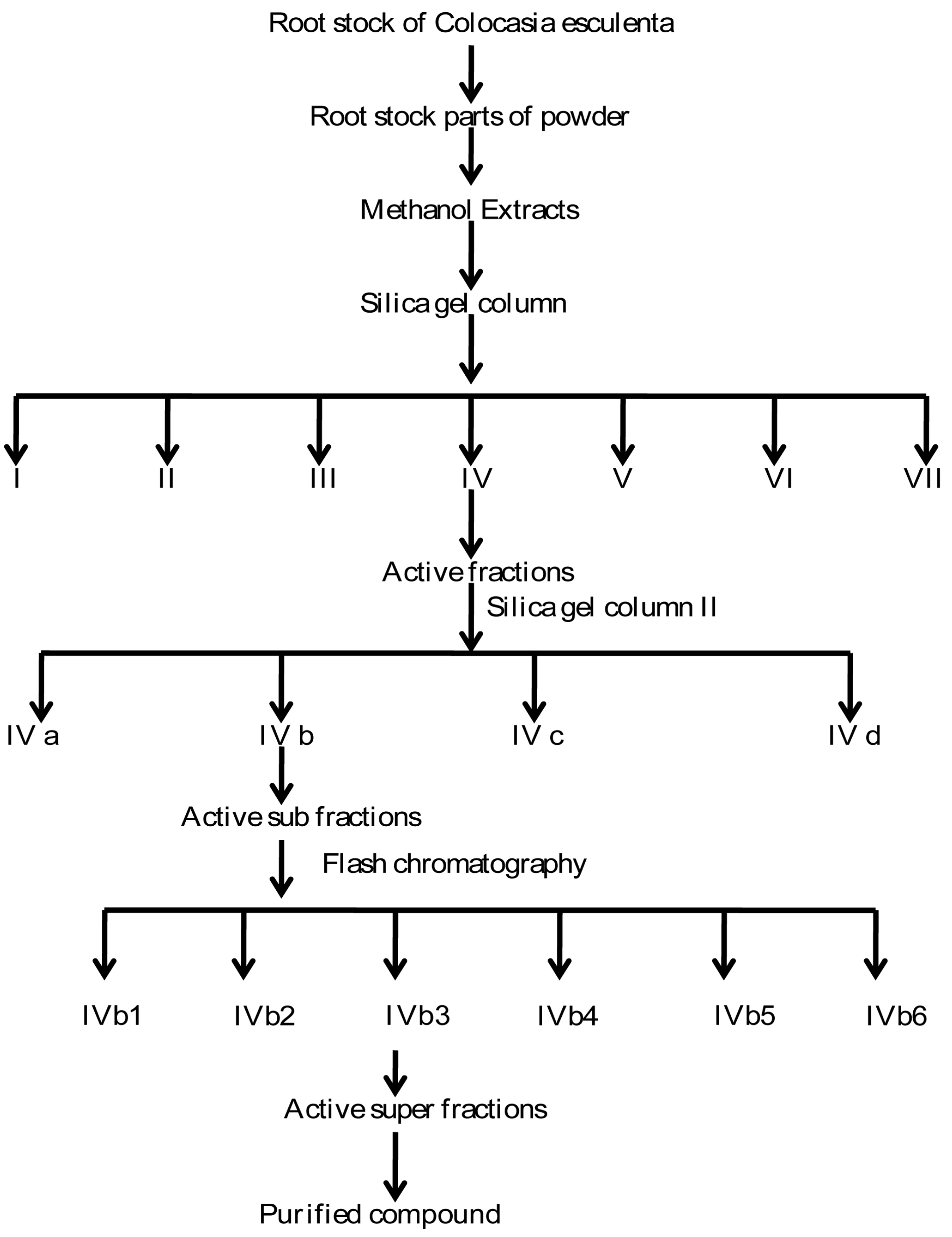

Figure S1: Schematic diagram for isolation for purified compound from methanol extract of *Colocasia esculenta*

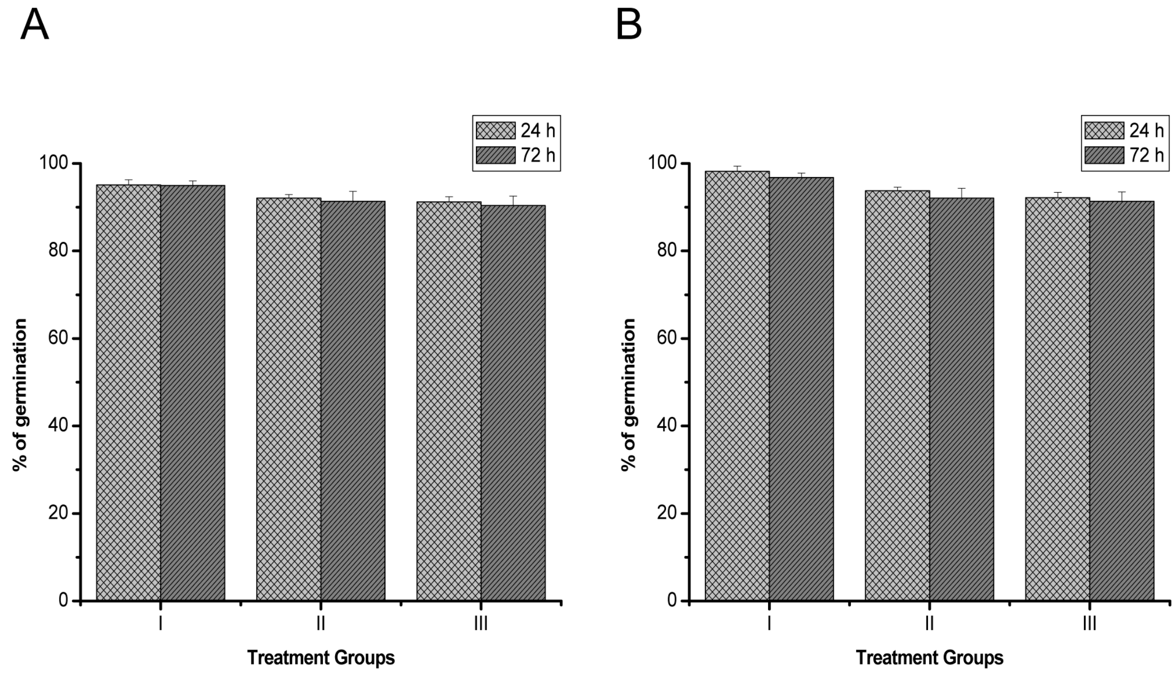

Figure S2| Effect of 2, 3-Dimethylmaleic anhydride on germination of A) wheat, and B) maize.

I) Control

II) 50 µg/l

III) 100 µg/l

Supplementary Methods

Isolation. The healthy mature root stocks of *C. esculenta* were collected from Imma market, Imphal and Bishnupur, Manipur, India. The root stock part was cut into small pieces, dried at 40 °C, and powdered. One hundred gram of root stock part powder was sequentially extracted with a series of solvents, viz., hexane, ethyl acetate, chloroform, acetone and methanol in a Soxhlet apparatus. Each individual extract was evaporated *in vacuo* and tested for insecticidal activity through biofumigation. Among them the methanolic extract showed maximum insecticidal activity (Fig. 1).

The methanolic extract (15.2 g) (from 250 gm) was subjected to column chromatography using a glass column (length; 50 cm, diameter: 3 cm) packed with silica gel (60-120 mesh) and eluted with hexane and ethyl acetate (100:0, I; 50:50, II; 25: 75, III) and followed by a stepwise gradient of ethyl acetate and methanol (100:0, IV; 50:50, V; 25:75, VI; 0:100, VII). Seven fractions of 300 ml each were collected, concentrated under reduced pressure, and assayed for insecticide activity. Fractions showing insecticidal activity were pooled into active fractions. The most active fraction (IV) showing insecticidal activity was separated into sub-fractions by chromatography using ethyl acetate and methanol with increasing polarity (100: 0, IVa; 25:75, IV b; 50:50 IV c and 0:100, IV d). The active compound responsible for insecticidal activity was finally isolated by silica gel column chromatography from the most active sub-fraction (IV b), by flash chromatography (column length 12 cm) using chloroform and methanol (75:25) as eluent. The purity of isolated biofumigant compound was checked by GCMS and the characterization done using spectroscopic techniques.

Gas chromatography-mass spectrometry. The purified compound from methanol extract of *C. esculenta* was analyzed by using GC-MS (7890 GC and 7200 QTOF respectively, Agilent Technologies) with HP-5 MS phenyl methyl siloxane non polar capillary column (0.25 mm x 30 m x0. 25 µm (19091S-433). Helium (99.999%) was used as a mobile phase. The mass spectra generated using the MS was compared with the Wiley mass spectral library (Wiley W9N11.L and NIST 2.0 version).

Fourier transformer infrared (FTIR) spectrometry. The FTIR spectrum was recorded using a Nicolet 5700 (Thermo electron, Madison, WI, USA) spectrometer at room temperature. The purified compound was dissolved in chloroform and scanned in the range of 400-4000 cm^{-1}.

^{1}H and ^{13}C Nuclear Magnetic Resonance Spectroscopy. Nuclear Magnetic Resonance (NMR) spectra were recorded on a Bruker DRX 500 NMR instrument operating at 400 MHz for ^{1}H and 100 MHz for ^{13}C at room temperature. A region from 0 to 12 ppm for ^{1}H and 0–200 ppm for ^{13}C was employed. Signals were referred to the internal standard tetramethylsilane. About 10 mg of the sample dissolved in 0.5 ml of $CDCl_3$ was used for recording the spectra.

Preparation of mixed-age-cultures. *T. castaneum* cultures were reared on whole wheat flour fortified with 5% dried yeast, *O. surinamensis* on a 5:5:1 mixture of broken wheat, rolled oats and yeast, whereas *S. oryzae* and *R. dominica* were reared on whole wheat. *R. dominica* cultures was maintained at 30±1°C and 70% r.h. and cultures of other insects were maintained at 25±1°C and 65±10% r.h. From these cultures, adults (1-2 weeks old) were taken for preparation of mixed-age cultures. About 300 adults of *T. castaneum* and *O. surinamensis* were released into 1 kg of respective culture medium in 2 liter glass jars-Similarly, 300 adults of *S. oryzae* and *R. dominica* were allowed to breed separately in 1 kg

of wheat in 2 litre glass jars. After one week, the adults were shifted from the cultures. A series of cultures of the respective species were thus maintained continuously. The insect culture jars were maintained at temperature 30 ± 1°C for *R. dominica* and 25 ± 1°C for other insects. Cultures in six successive weeks (containing 5-6, 4-5, 3-4, 2-3, 1-2, and 0-1 week old insects) were pooled such that the pooled populations contained all developmental stages of the respective species. The pooled mixture of individual species served as mixed-age cultures for toxicity tests.

Rearing media containing mixed-age cultures of individual species was weighed separately in 50 g aliquots and transferred into cloth bags of size 20 cm x 14 cm. These cloth bags were placed individually in 0.85 L desiccators that served as the fumigation chambers. The desiccators were provided with holes sealed by rubber septa for injecting 2,3-dimethylmaleic anhydride.

Fumigation. The insects were exposed to a range of doses of 2,3-dimethylmaleic anhydride dissolved in methanol (0.3 - 50 µg/ L) for 24 h and 72 h at 26 ± 2° C. For each species, there were 5 replicates per dose with an equal number of untreated control replicates. Gas-tight micro syringes were used for injecting the fumigant. After 24 h and 72 h, the gas in the desiccator was released by opening the lids of the desiccator and leaving for aeration. The mortality of insects was examined.

Post-Fumigation Operations. At the end of exposure, the test insect bags were taken out of the desiccators. The contents of the bags were transferred to individual bottles (12 cm x 5 cm size) and kept at the rearing temperature and humidity conditions for 8 weeks. The insects (*S. oryzae* and *R. dominica*) which emerged from wheat or survived as adults (*T. castaneum, C. ferrugineus* and *O. surinamensis*) in their respective media were checked at

weekly intervals for 8 weeks. Similarly, counts were made in untreated control batches every week. Percent kill was determined by taking the survival/emergence in the controls as 100%. In each bioassay mortality was recorded and insects that did not move when lightly probed or shaken in light and mild heat were considered dead.
